# Supplementary material for: Complexome profiling of the Chlamydomonas psb28 mutant reveals TEF5 as an early PSII assembly factor
Source: Plant Cell. 2025 Mar 17;37(6):koaf055. doi: 10.1093/plcell/koaf055 (PMC12164590; doi:10.1093/plcell/koaf055)
Supplement: koaf055_Supplementary_Data [file koaf055_supplementary_data.zip › psb28_interactive.zip!/plots/intensity/qualityPlots/peptides/PRPL10_Cre06.g272850.t1.2.html]

### Cre06.g272850.t1.2

Trivial name: PRPL10  
SeqCoverage [median]: 23.80  
Mass: 25.90 kDa  
  
 

| Sequence | MissedCleavage | Mass [Da] | wtIntensity | muIntensity | Score |
| --- | --- | --- | --- | --- | --- |
| FKGLDVPTVQK | 1 | 1230.70 | 219.80 | 0.00 | 38.63 |
| ILPANTSVYVTK | 0 | 1304.73 | 0.00 | 357.74 | 53.68 |
| STVVFGLR | 0 | 877.50 | 688.87 | 0.00 | 65.80 |
| VAVSQTK | 0 | 731.42 | 265.52 | 360.57 | 81.38 |
| VSELDDDKTK | 1 | 1148.56 | 405.60 | 437.89 | 48.44 |
|  |  |  | 1579.8 | 1156.2 |  |
